# Hematologic Risk Factors for the Development of Retinopathy of Prematurity—A Retrospective Study

**DOI:** 10.3390/children10030567

**Published:** 2023-03-16

**Authors:** Gabriela Ildiko Zonda, Raluca Mogos, Alina-Sînziana Melinte-Popescu, Ana-Maria Adam, Valeriu Harabor, Dragos Nemescu, Demetra Socolov, Anamaria Harabor, Marian Melinte-Popescu, Maura Adelina Hincu, Ingrid-Andrada Vasilache, Alexandru Carauleanu, Gigi Adam, Luminita Paduraru

**Affiliations:** 1 Department of Mother and Child Care, “Grigore T. Popa” University of Medicine and Pharmacy, 700115 Iasi, Romania; 2 Department of Mother and Newborn Care, Faculty of Medicine and Biological Sciences, ‘Ștefan cel Mare’ University, 720229 Suceava, Romania; 3 Clinical and Surgical Department, Faculty of Medicine and Pharmacy, ‘Dunarea de Jos’ University, 800216 Galati, Romania; 4 Department of Internal Medicine, Faculty of Medicine and Biological Sciences, ‘Ștefan cel Mare’ University, 720229 Suceava, Romania; 5 Department of Pharmaceutical Sciences, Faculty of Medicine and Pharmacy, ‘Dunarea de Jos’ University, 800216 Galati, Romania

**Keywords:** retinopathy of prematurity, platelet mass index, risk factor identification, hematological parameters

## Abstract

(1) Background: Retinopathy of prematurity (ROP) can cause severe visual impairment or even blindness. We aimed to assess the hematological risk factors that are associated with different stages of ROP in a cohort of preterm newborns, and to compare the clinical characteristics and therapeutic interventions between groups. (2) Methods: This retrospective study included 149 preterm newborns from a tertiary maternity hospital in Romania between January 2018 and December 2018, who were segregated into: Group 1 (with ROP, *n* = 59 patients), and Group 2 (without ROP, *n* = 90 patients). The patients that were affected by ROP were subsequently divided into the following subgroups: Subgroup 1 (Stage 1, *n* = 21), Subgroup 2 (Stage 2, *n* = 35), and Subgroup 3 (Stage 3, *n* = 25). The associations were analyzed using multivariate logistic regression and sensitivity analysis. (3) Results: Platelet mass indexes (PMI) that were determined in the first, seventh, and tenth days of life were significantly associated with Stage 1 ROP. PMI determined in the first day of life was also significantly associated with Stage 2 ROP. The sensitivity and specificity of these parameters were modest, ranging from 44 to 57%, and 59 to 63%. (4) Conclusions: PMI has a modest ability to predict the development of ROP.

## 1. Introduction

Retinopathy of prematurity (ROP) is a proliferative retinal vascular disorder that can determine important visual impairment or even blindness. Despite being a preventable disease, it is estimated that approximately 50,000 children worldwide have lost their vision due to this disease [1]. Advances in prenatal care, as well as an increase in the number of neonatal intensive care units, have resulted in higher survival rates for preterm and low birth weight newborns. As a result, the number of children that are at risk of developing ROP has been growing [2]. The estimated incidence of this condition varies between 1.2% and 13.1%, depending on the examined geographic regions in recent reports [3,4,5,6].

ROP has a multifactorial etiology, with premature birth, low birth weight, and hyperoxia being the most frequently cited risk factors [2,7]. Many other factors, including hyperglycemia, genetic factors, sepsis, bronchopulmonary dysplasia, and intraventricular hemorrhage have been linked to ROP development [8,9]. While they are recognized to have a role, the exact pathogenesis of retinopathy of prematurity is unclear; instead, several variables could be contributing to its unique etiology and progression.

ROP pathogenesis is divided into two stages: the first stage begins immediately after birth, when alterations in retinal vascularization are brought about by the suppression of vascular endothelial growth factor (VEGF) induced by artificial hyperoxygenation. The second stage starts at 32 weeks of gestation, when the avascular retina triggers a pathological rise in VEGF levels, leading to aberrant retinal vessel proliferation [10,11,12].

Several biomarkers, such as metabolites, cytokines, growth factors, non-coding RNAs, gut microbiota, or oxidative stress markers, have been proposed for the diagnosis or prediction of ROP [13]. The role of thrombocyte parameters in the prediction of ROP has been studied in several papers that have outlined the involvement of these elements in neoangiogenesis [14]. Thrombocytopenia, mean platelet volume (MPV), and platelet mass index (PMI) have been proven to be promising biomarkers for the prediction of ROP in various studies [15,16,17].

Screenings for ocular diseases such as ROP often include an examination of the fundus via indirect ophthalmoscopy by a qualified ophthalmologist [18]. The ICROP3 is the International Classification of Retinopathy of Prematurity that should be used for a diagnosis of ROP. There are five stages of the disease, beginning with a line and progressing through a raised ridge, a vascularized ridge, and finally partial and full retinal detachment in stages 4 and 5, respectively.

ROP is currently treated mostly with laser photocoagulation and ablative cryotherapy. These treatments are effective because they eliminate the avascular retina, which serves as the source of the growth factors that cause new blood vessel formation [12,19]. Although these therapies may help lower the rate of new cases of blindness, they also come with risks such as inflammation, myopia, peripheral vision loss, and scar formation [20,21]. On the other hand, in recent years, anti-VEGF drugs, such as ranibizumab, bevacizumab, and aflibercept that are now available on the market have been progressively employed to down-regulate the overactive signaling pathway during the initial proliferative phase of retinopathy of prematurity [22,23]. Nevertheless, there is a lack of information on drug selection and dose, and the long-term effects on the eye and the human organism are unknown [24].

Clinical factors of ROP in neonates are poorly understood at present. The purpose of this research was to examine the clinical features and treatment interventions of ROP patients and controls, as well as to retrospectively analyze the hematological risk factors that are associated with various phases of ROP in a cohort of preterm neonates.

## 2. Materials and Methods

Over the period of January 2018 through December 2018, the ROP patients that were admitted to a Level III newborn critical care unit at the Clinical Hospital of Obstetrics and Gynecology “Cuza-Voda”, Iasi, Romania, were analyzed in this observational, retrospective, unicentric research. The Institutional Ethics Committee of the local hospital gave their permission for this research (No. 14181/25 October 2022). The parents or guardians of the infants that were included in the trial provided written informed consent. All procedures were performed in compliance with applicable regulations and standards.

All infants that were diagnosed with ROP at our tertiary care center within the aforementioned time frame who were delivered before 33 weeks of gestation were included; infants whose mothers were unable to give informed consent or whose medical records were incomplete were excluded.

Information was gathered via a systematic assessment of the hospital data of 149 newborns. Documentation was kept of the patients’ clinical characteristics (e.g., gestational age, birthweight, gender, Apgar score at 1 and 5 min), risk factors for ROP, antenatal administration of corticosteroids, hematological parameters recorded on various occasions (e.g., day 1, day 7, and postmenstrual weeks 32, 33, and 34), and diagnostic and therapeutic approaches. A comprehensive ophthalmological examination that was done at the regional hospital in accordance with a partnership agreement served as the foundation for confirming the clinical diagnosis that had been established earlier.

Group 1 consisted of newborns that were diagnosed with ROP (*n* = 59), whereas Group 2 included those who did not have the condition (*n* = 90). Subgroup 1 (Stage 1, *n* = 21), Subgroup 2 (Stage 2, *n* = 35), and Subgroup 3 (Stage 3, *n* = 25) were devised from the ROP patients using the ICROP3 classification [25]. These three stages corresponded to an acute phase of the disease. We did not record Stage 4 or 5 ROP.

Univariate statistical analysis was performed using Chi-squared and Fisher’s exact tests for categorical variables, and *t*-tests for continuous variables. Using an ANOVA followed by the Bonferroni post hoc test, it was determined whether or not there is a statistically significant difference between the subgroups in terms of their paraclinical features. The statistical analyses were carried out with the help of STATA SE software (version 17, 2022, StataCorp LLC, College Station, TX, USA).

In the multivariate analysis, we evaluated the association of individual hematological parameters with different stages of ROP using multinomial logistic regression. Those parameters who reached statistical significance (*p* < 0.05) were further evaluated using a sensitivity analysis.

## 3. Results

Group 1 consisted of 59 patients with a mean gestational age at delivery and standard deviation of 27.97 ± 2.50 weeks of gestation and Group 2 consisted of 90 patients with a mean gestational age at delivery and standard deviation of 29.83 ± 1.71 weeks of gestation (*p* < 0.001). (Table 1). Birth weight was considerably lower in infants who went on to develop ROP (1102.8 ± 379.52 vs. 1366.83 ± 319.92 g, *p* < 0.001) compared to the control group. There was also a statistically significant difference between their 1- and 5-min Apgar scores and those of the control group (*p* < 0.001).

Neonatal comorbidities are comparatively presented in Table 2 for the main groups. Neonates who developed ROP presented with significantly more intrauterine growth restriction (*p* = 0.04), mild bronchopulmonary dysplasia (*p* = 0.01), systemic infection (*p* < 0.001), and intraventricular hemorrhage (*p* = 0.004).

Therapeutic interventions that were applied to preterm neonates are comparatively presented in Table 3 for the main groups. Neonates who developed ROP have received significantly longer therapies such as high-flow oxygen, CPAP, and mechanical ventilation (*p* < 0.001). Moreover, transfusions of packed red blood cells were administered significantly more frequently to the ROP group in all the evaluated time frames (*p* < 0.05).

A comparison of the hematological parameters for the evaluated subgroups based on ANOVA analysis with a Bonferroni post hoc test is presented in Table 4. We could determine that between the evaluated subgroups there is an important variance regarding the following hematological parameters: (a) hemoglobin, repeatedly determined in the first ten days of life and at 34 postmenstrual weeks (*p* < 0.05); (b) hematocrit, repeatedly determined in the first ten days of life and at 34 postmenstrual weeks (*p* < 0.05); and (c) platelet mass index (PMI) determined in the first day of life (*p* = 0.023).

The associations between individual hematologic parameters and different stages of ROP were determined using multinomial logistic regression (Table 5, Table 6 and Table 7). PMI determined in the first (odds ratio/OR: 4.15; 95% confidence interval/CI: 1.39–7.50; *p* = 0.032), seventh (OR: 3.57; 95% CI: 0.65–10.05; *p* = 0.023), and tenth days of life (OR: 3.72; 95% CI: 0.46–8.13; *p* = 0.018) were significantly associated with Stage 1 ROP. PMI determined in the first day of life (OR: 7.67; 95%CI: 1.87–16.48; *p* = 0.036) was also significantly associated with Stage 2 ROP, while none of the evaluated parameters were associated with ROP 3.

**Figure 1:**
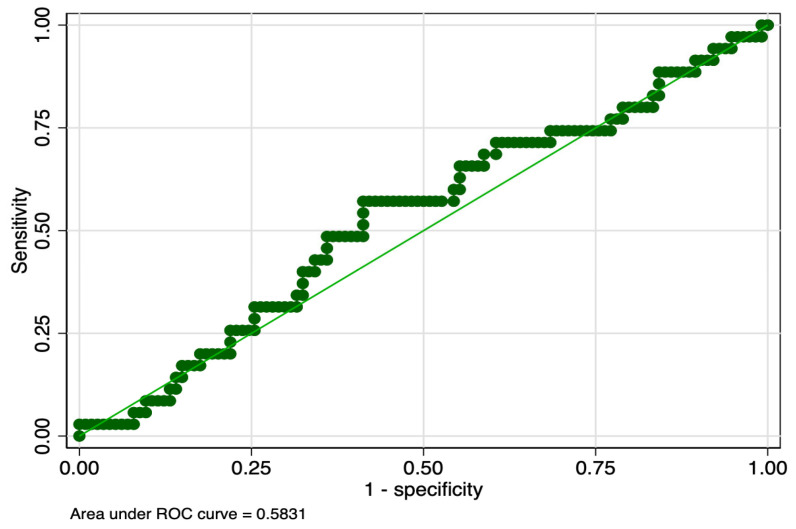
ROC curve of PMI determined in the first day of life for the prediction of Stage 1 ROP. Legend: ROC—receiver operating characteristic; PMI—platelet mass index; ROP—retinopathy of prematurity.

**Figure 2:**
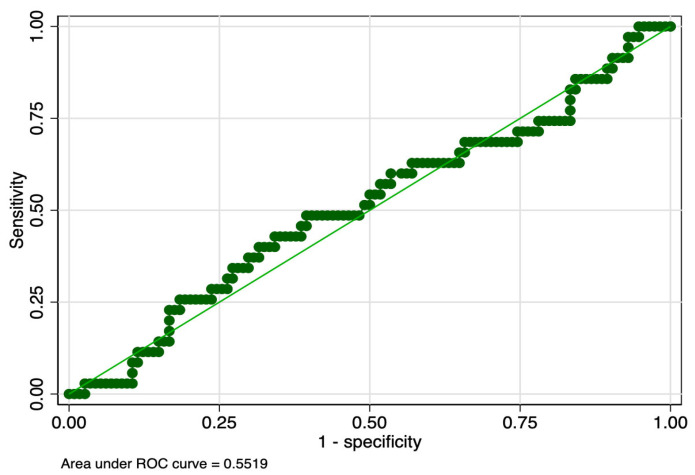
ROC curve of PMI determined in the seventh day of life for the prediction of Stage 1 ROP. Legend: ROC—teceiver operating characteristic; PMI—platelet mass index; ROP—retinopathy of prematurity.

**Figure 3:**
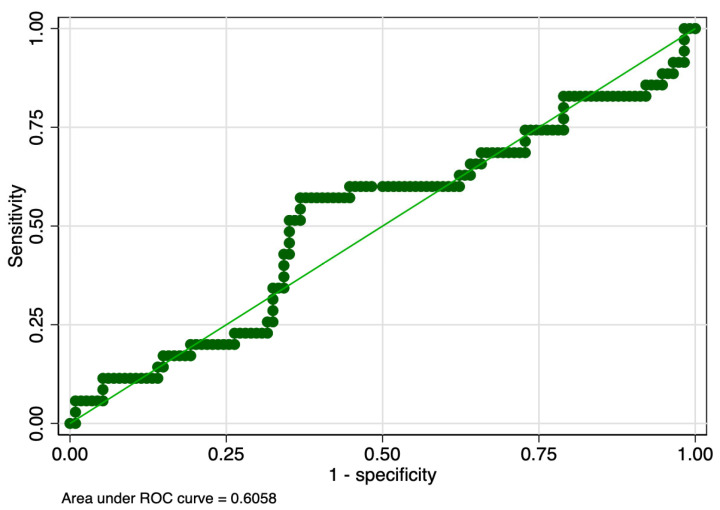
ROC curve of PMI determined in the tenth day of life for the prediction of Stage 1 ROP. Legend: ROC—receiver operating characteristic; PMI—platelet mass index; ROP—retinopathy of prematurity.

**Figure 4:**
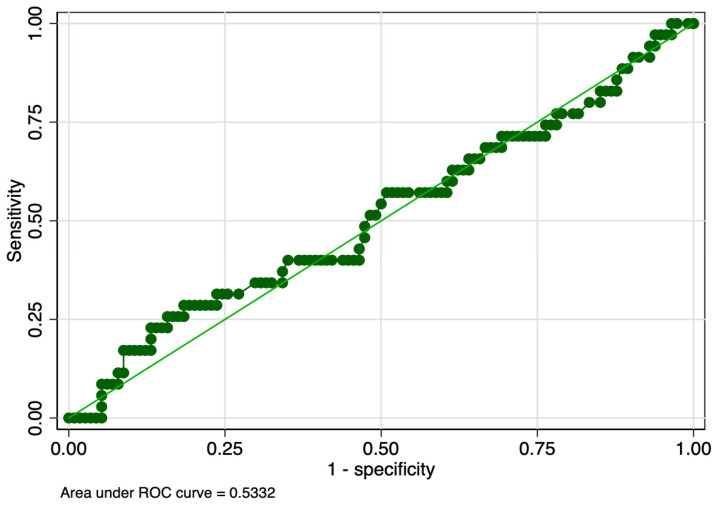
ROC curve of PMI determined in the first day of life for the prediction of Stage 2 ROP. Legend: ROC—receiver operating characteristic; PMI—platelet mass index; ROP—retinopathy of prematurity.

The sensitivity analysis revealed that PMI determined in the first and tenth days of life had equal sensitivity (57%), but the latter had higher specificity (63% versus 59%), and ROC value (0.60 versus 0.58) (Table 8). PMI determined in the first day of life had slightly lower sensitivity (44% versus 57%), but higher specificity (61% versus 59%) between stages 2 and 1 of ROP. Graphic representations of ROC curves correspondent to the analyzed parameters are presented in Figure 1, Figure 2, Figure 3 and Figure 4.

## 4. Discussion

In this retrospective study, we assessed the hematological risk factors that are associated with ROP in a cohort of preterm newborns from Romania, and we comparatively analyzed the clinical characteristics and therapeutic interventions between controls and ROP patients. Our univariate analyses indicated that newborns who later developed ROP had significantly lower birthweight and Apgar scores at 1 and 5 min compared with the control group. Moreover, neonates who developed ROP presented with significantly more intrauterine growth restriction, mild bronchopulmonary dysplasia, systemic infection, and intraventricular hemorrhage.

Indeed, IUGR is a known factor for ROP, and a recent study retrospective cohort study by Chu et al. demonstrated that IUGR infants were more likely to have a worse stage of ROP and treatment-requiring ROP compared to non-IUGR infants [26]. Additionally, it was demonstrated that a low 5-min Apgar score and an Apgar score of 6 or less at 5 min were significant risk factors for the manifested ROP to progress to stages requiring treatment [27]. Both bronchopulmonary dysplasia and ROP have a multifactorial determinism, intertwining various defective angiogenic and inflammatory mechanisms [28]. Intraventricular hemorrhage and necrotizing enterocolitis are also two disorders that are strongly linked to ROP [29,30,31], but we could not determine a significantly higher incidence of necrotizing enterocolitis in the ROP group compared with the controls (*p* = 0.30).

Our results showed that neonates who developed ROP had received significantly longer therapies such as high-flow oxygen, CPAP, and mechanical ventilation. It was shown that premature newborns have different oxygen necessities at different postnatal ages, and that each gestational age category has an optimal range for oxygen saturation threshold [32]. Prolonged oxygen therapy and maintenance of an inadequate oxygen saturation can lead to ROP.

Two studies investigated the possibility that higher oxygen saturation thresholds (96–99% and 95–98%) in newborns with ROP who still required supplemental oxygen at 32 weeks of gestation would be advantageous [33,34]. A greater oxygen saturation goal was related with poorer respiratory outcomes in both investigations, and neither study found any substantial advantage from setting a higher target. A recent epidemiological research that analyzed ROP trends in the USA also found a favorable association between the severity of ROP and the usage of supplemental oxygen [30].

Transfusions of packed red blood cells were administered significantly more frequent to the ROP group in all the evaluated time frames. A recent systematic review and meta-analysis evaluated the relationship between red blood cells transfusion and the development of ROP, demonstrating that red blood cells transfusion is an independent risk factor for the development of ROP (OR = 1.50, 95% CI: 1.27–1.76), especially in younger preterm infants (OR = 1.77, 95% CI: 1.29–2.43) [35].

Our analysis showed that PMI determined in the first, seventh, and tenth days of life were significantly associated with Stage 1 ROP. PMI determined in the first day of life was also significantly associated with Stage 2 ROP, while none of the evaluated parameters were associated with ROP 3. However, our sensitivity analysis showed only modest results for these parameters, with sensitivity ranging from 44 to 57%, and specificity ranging from 59 to 63% for each parameter. Even though the PMI values that were determined in the first 10 days of life appeared to be significantly associated with the development of Stage 1 and 2 ROP, based on our sensitivity analysis results, we do not recommend using the hematological parameters for the early prediction of ROP.

Similar results were obtained in a retrospective study that analyzed the contribution of thrombocyte parameters, including thrombocyte count, presence of thrombocytopenia, mean platelet volume, platelet distribution width (PDW), and platelet mass index, to the ROP development. The study included 120 preterm infants segregated into three groups: Group 1- infants who developed type-1 ROP and received treatment; Group 2- infants who developed ROP and were not treated for ROP; and Group 3- infants who did not develop ROP. The results did not show a statistically significant difference between the evaluated groups regarding the evaluated thrombocyte parameters [14].

On the other hand, a few studies demonstrated that PMI can be considered a marker for the prognosis of type 2 ROP. Korkmaz et al. investigated the PMI’s potential to predict the need for laser photocoagulation in preterm newborns that are at risk of developing ROP [15]. The PMI values, determined at the 32nd postmenstrual week, considered to reflect the second phase of ROP, had an AUC value of 0.63, with a sensitivity of 60%, and a specificity of 68% for the predicted outcome.

Our study’s limited sample size is one of its limitations since it may indicate selection bias. Another limitation is that the study was carried out using a retrospective design; we believe that a prospective strategy might provide more convincing evidence linking certain risk factors with the ROP’s development. The results of this study could also be affected by a selection bias resulting from an imbalanced sex ratio of premature infants in the study group. Lastly, the variability of the clinical and paraclinical findings constitutes a limitation in the relationship with the above-mentioned caveats. A more comprehensive understanding of the issue might be obtained from studies on larger cohorts of patients recruited from multiple centers.

Specific risk factor identification in preterm infants with high risk of developing ROP could allow an individualized patient management, and could constitute an argument for the neonatologists in favor of the best therapeutic decisions. Moreover, these risk factors for ROP progression could be presented to parents during the counseling sessions in order to offer them a comprehensive perspective on the ROP clinical evolution.

More effort should be put into developing new strategies for the prediction and prevention of retinopathy of prematurity, considering the worldwide epidemiological burden. Adjusting the supplementary oxygen thresholds for preterm newborns and early administration of breast milk constitute key elements for preventing ROP progression. Moreover, the identification of this debilitating disease in the early stages would allow clinicians to offer various therapeutic strategies for the affected newborns, and improve the overall outcome.

## Figures and Tables

**Table 1:** Clinical characteristics of the main groups.

Parameter	Group 1 (with ROP, *n* = 59 Patients)	Group 2 (without ROP, *n* = 90 Patients)	*p* Value
Gestational age at birth, weeks (mean ± SD)	27.97 ± 2.50	29.83 ± 1.71	<0.001
Birth weight, grams (mean ± SD)	1102.8 ± 379.52	1366.83 ± 319.92	<0.001
Newborns’ gender (*n*/%)	Male = 30 (50.8%) Female = 29 (49.2%)	Male = 56 (62.2%) Female = 34 (37.8%)	0.16
Apgar score at 1 min (mean ± SD)	4.49 ± 1.66	5.42 ± 1.59	<0.001
Apgar score at 5 min (mean ± SD)	5.64 ± 1.39	6.47 ± 1.30	<0.001
Antenatal corticosteroid administration (*n*/%)	Yes = 12 (20.3%)	Yes = 13 (14.4%)	0.37

Table legend: ROP—retinopathy of prematurity; SD—standard deviation.

**Table 2:** Neonatal comorbidities in the main groups.

Neonatal Comorbidities	Group 1 (with ROP, *n* = 59 Patients)	Group 2 (without ROP, *n* = 90 Patients)	*p* Value
NEC (*n*/%)	Yes = 11 (18.6%)	Yes = 12 (13.3%)	0.30
IUGR (*n*/%)	Yes = 14 (23.7%)	Yes = 10 (11.1%)	0.04
Mild BPD (*n*/%)	Yes = 10 (16.9%)	Yes = 4 (4.4%)	0.01
Moderate BPD (*n*/%)	Yes = 5 (8.5%)	Yes = 4 (4.4%)	0.31
Severe BPD (*n*/%)	Yes = 3 (5.1%)	Yes = 2 (2.2%)	0.34
Systemic infection (*n*/%)	Yes = 18 (30.5%)	Yes = 4 (4.4%)	<0.001
IVH (*n*/%)	Yes = 28 (47.5%)	Yes = 22 (24.4%)	0.004

Table legend: ROP—retinopathy of prematurity; NEC—necrotizing enterocolitis; IUGR—intrauterine growth restriction; BPD—bronchopulmonary dysplasia; IVH—intraventricular hemorrhage.

**Table 3:** Therapeutic interventions in the main groups.

Therapeutic Interventions	Group 1 (with ROP, *n* = 59 Patients)	Group 2 (without ROP, *n* = 90 Patients)	*p* Value
FiO2 in delivery room (mean ± SD)	0.42 ± 0.22	0.39 ± 0.19	0.16
High-flow oxygen therapy, days (mean ± SD)	11.83 ± 20.01	2.87 ± 8.54	<0.001
CPAP, days (mean ± SD)	8.80 ± 8.59	4.10 ± 4.51	<0.001
CPAP FiO_2_ > 30%, days (mean ± SD)	0.46 ± 2.02	0.04 ± 0.20	<0.001
CPAP FiO_2_: 25–30%, days (mean ± SD)	0.93 ± 1.57	0.33 ± 1.06	<0.001
CPAP FiO_2_: 21–25%, days (mean ± SD)	7.32 ± 7.85	3.74 ± 3.87	<0.001
Mechanical ventilation, days (mean ± SD)	14.34 ± 22.87	2.82 ± 6.53	<0.001
Transfusion of packed red blood cells <7 days (*n*/%)	Yes = 23 (39%)	Yes = 11 (12.2%)	<0.001
Transfusion of packed red blood cells days 7–28 (*n*/%)	Yes = 27 (45.8%)	Yes = 19 (21.1%)	0.001
Transfusion of packed red blood cells >28 days (*n*/%)	Yes = 38 (64.4%)	Yes = 34 (37.8%)	0.001
Erythropoietin administration (*n*/%)	Yes = 55 (42%)	Yes = 76 (58%)	0.10
Oral iron administration	Yes = 59 (100%)	Yes = 82 (91.1%)	0.019

Table legend: ROP—retinopathy of prematurity; FiO_2_—the fraction of inspired oxygen; CPAP—continuous positive airway pressure; SD—standard deviation.

**Table 4:** Comparison of hematological parameters for the patients that were included in the analyzed subgroups.

Hematological Parameter	Subgroup 1 (Stage 1, *n* = 35)	Subgroup 2 (Stage 2, *n* = 18)	Subgroup 3 (Stage 3, *n* = 6)	Control Group (*n* = 90)	Mean Squares between Groups	F Score	*p*-Value
Hb, number ×10^6^/mm^3^, day 1 (mean ± SD)	15.33 ± 3.19	15 ± 2.82	14.51 ± 3.86	16.38 ± 2.22	19.40	2.82	0.041
Ht, %, day 1 (mean ± SD)	48.31 ± 10.14	47.05 ± 8.22	45.65 ± 11.49	51.78 ± 6.84	217.751	3.329	0.021
PLT, number/mm^3^ (mean ± SD)	246.628 ± 100.699	248.666 ± 107.182	340.666 ± 171.011	235.122 ± 73.8692	21,260.477	2.630	0.052
MPV, fL, day 1 (mean ± SD)	6.44 ± 1.11	5.97 ± 0.83	6.88 ± 1.65	6.33 ± 1.20	1.50	1.11	0.347
PMI, fL*nL^−1^, day 1 (mean ± SD)	1546.82 ± 615.66	1448.16 ± 589.68	2148.2 ± 791.36	1460.04 ± 466.55	927,678.04	3.25	0.023
Hb, number ×10^6^/mm^3^, day 7 (mean ± SD)	14.02 ± 2.70	14.14 ± 2.74	13.6 ± 4.03	15.35 ± 2.30	21.660	3.369	0.020
Ht, %, day 7 (mean ± SD)	43.56 ± 8.45	44.38 ± 7.91	43.25 ± 13.03	48.56 ± 6.97	274.203	4.581	0.004
PLT day 7, number/mm^3^ (mean ± SD)	236.314 ± 99.170	263.277 ± 123.695	306.666 ± 143.960	235.533 ± 83.622	12,711.481	1.396	0.246
MPV, fL, day 7 (mean ± SD)	6.92 ± 1.52	6.76 ± 1.26	7.13 ± 3.27	6.49 ± 1.24	2.129	1.038	0.378
PMI, fL*nL^−1^, day 7 (mean ± SD)	1563.45 ± 556.35	1677.75 ± 696.29	1894.91 ± 569.40	1502.09 ± 553.85	406,212.909	1.235	0.299
Hb, number ×10^6^/mm^3^, day 10 (mean ± SD)	13.28 ± 2.27	12.92 ± 2.70	11.25 ± 2.56	14.52 ± 2.21	35.853	6.740	<0.001
Ht, %, day 10 (mean ± SD)	40.91 ± 7.04	40.39 ± 8.18	35.31 ± 8.24	45.18 ± 6.65	350.064	7.140	<0.001
PLT day 10, number/mm^3^ (mean ± SD)	310.228 ± 120.888	311.28 ± 167.19	243.33 ± 98.22	308.09 ± 107.35	8354.098	0.592	0.621
MPV, fL, day 10 (mean ± SD)	7.43 ± 1.58	7.84 ± 2.07	7.63 ± 2.58	7.39 ± 1.45	1.068	0.407	0.748
PMI, fL*nL^−1^, day 10 (mean ± SD)	2273.29 ± 957.03	2332.27 ± 1214.06	1671.51 ± 460.78	2252.51 ± 843.05	714,409.469	0.860	0.464
Hb, number ×10^6^/mm^3^, week 32 (mean ± SD)	10.89 ± 2.45	10.51 ± 2.04	10.16 ± 1.29	11.06 ± 2.43	2.808	0.502	0.682
Ht, %, week 32 (mean ± SD)	33.96 ± 7.62	32.60 ± 6.46	31.80 ± 4.68	37.23 ± 26.29	193.581	0.436	0.727
PLT week 32, number/mm^3^ (mean ± SD)	397.81 ± 152.65	394.55 ± 164.39	421.33 ± 243.62	418.51 ± 125.37	5555.259	0.273	0.845
MPV, fL, week 32 (mean ± SD)	6.94 ± 1.09	7.26 ± 1.66	7.33 ± 1.99	7.17 ± 1.73	0.678	0.262	0.853
PMI, fL*nL^−1^, week 32 (mean ± SD)	2711.19 ± 1017.06	2731.77 ± 1022.69	3289.08 ± 2369.87	2951.12 ± 959.16	950,540.143	0.846	0.471
Hb, number ×10^6^/mm^3^, week 33 (mean ± SD)	10.62 ± 2.67	10.14 ± 1.49	8.11 ± 4.11	10.53 ± 2.08	11.910	2.296	0.080
Ht, %, week 33 (mean ± SD)	33.06 ± 7.98	31.75 ± 3.99	26.40 ± 13.86	32.91 ± 6.44	86.426	1.767	0.156
PLT week 33, number/mm^3^ (mean ± SD)	354.74 ± 133.31	356.06 ± 139.80	324.33 ± 235.03	392.61 ± 114.43	21,260.173	1.296	0.278
MPV, fL, week 33 (mean ± SD)	6.87 ± 1.72	6.66 ± 1.14	5.78 ± 3.07	6.83 ± 1.30	2.265	1.018	0.387
PMI, fL*nL^−1^, week 33 (mean ± SD)	2474.70 ± 908.27	2339.45 ± 880.26	2262.20 ± 1637.63	2626.48 ± 760	669,691.404	0.916	0.435
Hb, number ×10^6^/mm^3^, week 34 (mean ± SD)	9.56 ± 3.29	9.29 ± 2.80	6.88 ± 5.59	10.04 ± 2.20	20.526	2.729	0.046
Ht, %, week 34 (mean ± SD)	29.46 ± 9.94	29.25 ± 8.77	21.31 ± 17.26	31.46 ± 6.85	216.777	3.038	0.031
PLT week 34, number/mm^3^ (mean ± SD)	331.40 ± 148.02	310.56 ± 129.10	281.50 ± 264.17	374.70 ± 128.60	40,603.549	2.066	0.107
MPV, fL, week 34 (mean ± SD)	6.49 ± 2.22	5.98 ± 1.77	4.93 ± 4.00	6.64 ± 2.02	6.989	1.521	0.212
PMI, fL*nL^−1^, week 34 (mean ± SD)	2312.79 ± 973.83	1923.92 ± 795.28	2180.01 ± 2268.03	2474.78 ± 905.95	1,626,865.633	1.664	0.177

Table legend: ROP—retinopathy of prematurity; Hb—haemoglobin; Ht—hematocrit; MPV—mean platelet volume; PMI—platelet mass index; SD—standard deviation.

**Table 5:** Multinomial logistic regression of individual hematological parameters for patients with ROP Stage 1.

Hematological Parameter	Odds Ratio	95%CI Lower Limit	95%CI Upper Limit	*p*-Value
Hb day 1	0.40	0.14	1.15	0.093
Hb day 7	1.32	0.95	1.84	0.090
Hb day 10	0.99	0.95	1.03	0.703
Hb week 32	0.97	0.21	4.51	0.975
Hb week 33	1.00	0.99	1.00	0.649
Hb week 34	3.36	0.84	13.38	0.085
Ht day 1	0.64	0.42	1.00	0.051
Ht day 7	1.01	0.98	1.05	0.314
Ht day 10	2.57	0.85	7.75	0.092
Ht week 32	0.99	0.99	1.00	0.347
Ht week 33	2.32	0.68	7.84	0.174
Ht week 34	0.70	0.46	1.06	0.093
PLT day 1	0.98	0.96	1.00	0.216
PLT day 7	0.43	0.16	1.14	0.090
PLT day 10	1.00	0.99	1.00	0.216
PLT week 32	1.15	0.74	1.78	0.518
PLT week 33	0.98	0.87	1.10	0.812
PLT week 34	1.00	0.99	1.02	0.351
MPV day 1	0.92	0.31	2.72	0.890
MPV day 7	0.99	0.99	1.00	0.253
MPV day 10	1.28	0.26	6.34	0.755
MPV week 32	0.95	0.56	1.59	0.852
MPV week 33	1.00	0.98	1.01	0.851
MPV week 34	1.188	0.55	2.56	0.658
PMI day 1	4.151	1.39	7.50	0.032
PMI day 7	3.57	0.65	10.05	0.023
PMI day 10	3.72	0.46	8.13	0.018
PMI week 32	0.99	0.98	1.01	0.839
PMI week 33	1.11	0.58	2.12	0.743
PMI week 34	1.00	0.99	1.00	0.594

Table legend: CI—confidence interval; Hb—haemoglobin; Ht—hematocrit; MPV—mean platelet volume; PMI—platelet mass index; SD—standard deviation.

**Table 6:** Multinomial logistic regression of individual hematological parameters for patients with ROP Stage 2.

Hematological Parameter	Odds Ratio	95%CI Lower Limit	95%CI Upper Limit	*p*-Value
Hb day 1	2.93	0.79	10.83	0.107
Hb day 7	1.05	0.33	3.32	0.932
Hb day 10	0.71	0.15	3.32	0.671
Hb week 32	2.90	0.67	4.12	0.089
Hb week 33	0.21	0.01	2.46	0.216
Hb week 34	0.21	0.01	3.00	0.252
Ht day 1	0.66	0.42	1.03	0.073
Ht day 7	1.01	0.69	1.49	0.930
Ht day 10	1.06	0.62	1.80	0.831
Ht week 32	0.44	0.17	1.11	0.083
Ht week 33	1.54	0.70	3.36	0.276
Ht week 34	1.72	0.72	4.08	0.215
PLT day 1	0.96	0.89	1.04	0.411
PLT day 7	1.04	0.97	1.11	0.213
PLT day 10	1.03	0.97	1.08	0.251
PLT week 32	1.01	0.98	1.03	0.297
PLT week 33	0.99	0.96	1.01	0.556
PLT week 34	1.02	0.99	1.05	0.116
MPV day 1	0.02	0.005	1.18	0.061
MPV day 7	1.79	0.50	6.04	0.174
MPV day 10	2.78	0.97	7.96	0.056
MPV week 32	2.09	0.63	6.89	0.222
MPV week 33	1.43	0.39	5.22	0.587
MPV week 34	1.00	0.99	1.01	0.450
PMI day 1	7.67	1.87	16.48	0.036
PMI day 7	0.99	0.98	1.00	0.270
PMI day 10	0.99	0.98	1.00	0.307
PMI week 32	0.99	0.99	1.00	0.385
PMI week 33	0.99	0.99	1.00	0.958
PMI week 34	0.99	0.98	1.00	0.067

Table legend: CI—confidence interval; Hb—haemoglobin; Ht—hematocrit; MPV—mean platelet volume; PMI—platelet mass index; SD—standard deviation.

**Table 7:** Multinomial logistic regression of individual hematological parameters for patients with ROP Stage 3.

Hematological Parameter	Odds Ratio	95%CI Lower Limit	95%CI Upper Limit	*p*-Value
Hb day 1	0.84	0.64	1.09	0.201
Hb day 7	0.83	0.61	1.13	0.240
Hb day 10	0.57	0.38	0.87	0.069
Hb week 32	0.84	0.56	1.26	0.423
Hb week 33	0.84	0.59	1.19	0.333
Hb week 34	0.87	0.65	1.17	0.363
Ht day 1	1.06	0.89	1.26	0.479
Ht day 7	1.12	0.94	1.34	0.189
Ht day 10	0.77	0.61	0.97	0.061
Ht week 32	0.97	0.82	1.14	0.751
Ht week 33	0.97	0.86	1.08	0.630
Ht week 34	0.94	0.85	1.04	0.259
PLT day 1	0.91	0.89	2.03	0.430
PLT day 7	1.01	0.99	1.02	0.090
PLT day 10	0.97	0.95	1.99	0.314
PLT week 32	1.00	0.99	1.01	0.651
PLT week 33	1.00	0.99	1.01	0.726
PLT week 34	1.00	0.99	1.01	0.694
MPV day 1	0.75	0.12	4.59	0.759
MPV day 7	1.62	0.27	9.70	0.595
MPV day 10	0.94	0.30	2.92	0.924
MPV week 32	0.58	0.19	1.71	0.326
MPV week 33	0.51	0.18	1.44	0.209
MPV week 34	1.00	0.99	1.00	0.078
PMI day 1	1.00	0.99	1.00	0.153
PMI day 7	0.99	0.98	1.00	0.050
PMI day 10	1.00	0.99	1.00	0.375
PMI week 32	1.00	0.99	1.00	0.662
PMI week 33	1.00	0.99	1.00	0.148
PMI week 34	0.84	0.64	1.09	0.201

Table legend: CI—confidence interval; Hb—haemoglobin; Ht—hematocrit; MPV—mean platelet volume; PMI—platelet mass index; SD—standard deviation.

**Table 8:** Sensitivity analysis of significant hematological parameters for patients with ROP Stage 1 and 2.

ROP Stage	Hematological Parameter	Cut-Off (fL*nL^−1^)	Sensitivity (%)	Specificity (%)	ROC Value
1	PMI day 1	1514.3	57	59	0.58
	PMI day 7	1627.6	49	61	0.55
	PMI day 10	2321.8	57	63	0.60
2	PMI day 1	1570.4	44	61	0.53

Table legend: ROP—retinopathy of prematurity; PMI—platelet mass index; SD—standard deviation.

## Data Availability

The data that are presented in this study are available on request from the corresponding author. The data are not publicly available due to local policies.
